# Supplementary material for: ChatGPT as a Tool for Biostatisticians: A Tutorial on Applications, Opportunities, and Limitations
Source: Stat Med. 2025 Oct 23;44(23-24):e70263. doi: 10.1002/sim.70263 (PMC12548020; doi:10.1002/sim.70263)
Supplement: Supplementary file 1 — Data S1. Supporting Information. [file SIM-44-0-s001.zip › Supplementary Materials Additional.zip!/3.1_Systematic_Reviews_and_Meta_aspects/3.1_Forest Plot - 4o - Chat 8.pdf]

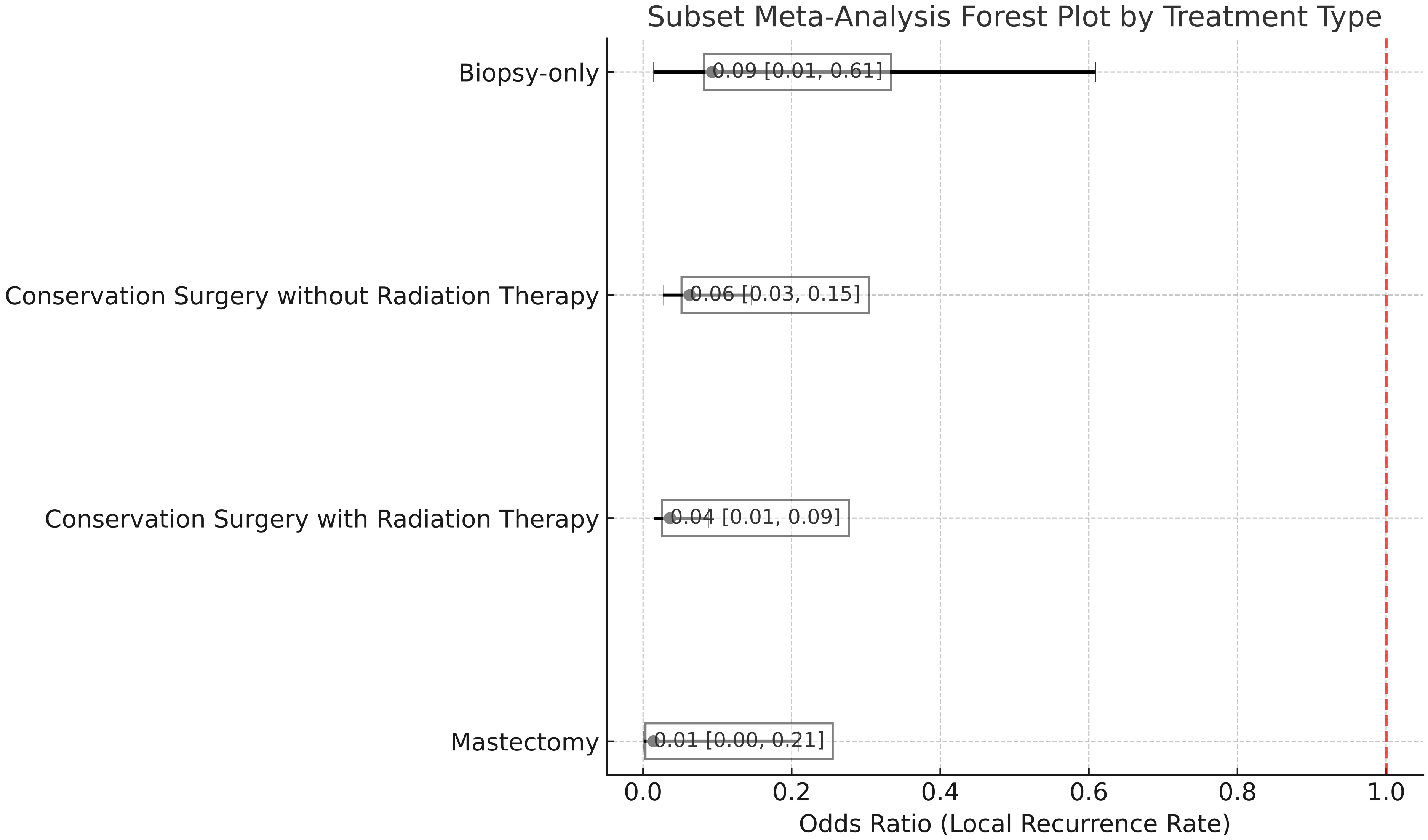
Subset Meta-Analysis Forest Plot by Treatment Type
Biopsy-only
Conservation Surgery without Radiation Therapy
Conservation Surgery with Radiation Therapy
Mastectomy
0.09 [0.01, 0.61]
0.06 [0.03, 0.15]
0.04 [0.01, 0.09]
0.01 [0.00, 0.21]
0.0
0.2
0.4
0.6
0.8
1.0
Odds Ratio (Local Recurrence Rate)
